# Outcomes of Patients Admitted to Psychiatry with First Presentation Psychosis and Hypothyroidism – a Retrospective Cohort Analysis

**DOI:** 10.1192/j.eurpsy.2026.12155

**Published:** 2026-07-31

**Authors:** D. Mackay, C. Hao, A. Lyer, J. King, S. Patten, E. Chan

**Affiliations:** 1 Psychiatry, University of Calgary; 2 Health Research Methods and Analytics, Alberta Health Services, Calgary; 3 Psychiatry, University of Ottawa, Ottawa, Canada

## Abstract

**Introduction:**

Psychotic symptoms occurring in the context of severe hypothyroidism, contemporarily referred to as myxedema psychosis (MP), have been described in numerous case reports. To date, no systematic primary research studies have been conducted to characterize MP within psychiatric inpatient populations.

**Objectives:**

We aimed to identify cases of MP using administrative data, determine its prevalence among first psychiatric admissions for psychosis, examine outcomes including risk of readmission, and explore the effect of antipsychotic treatment.

**Methods:**

We conducted a retrospective cohort analysis, using provincial administrative databases, of patients admitted for first-presentation psychosis between January 1, 2012, and March 31, 2022. Cases of MP were defined by elevated thyroid-stimulating hormone (TSH) at admission across thresholds of ≥40, ≥60, and ≥80 mIU/L, with analyses repeated excluding patients ≥60 years of age. Patients with a first psychiatric admission with psychosis and normal TSH were used as controls. Cox proportional hazards models were used to compare hazards of readmission. Within the MP cohort, we examined the association of antipsychotic treatment with readmission risk.

**Results:**

The results of survival analyses using different TSH thresholds (with or without an age threshold) are shown in Table 1. At a TSH threshold of ≥60 mIU/L and age <60, MP cases had a significantly reduced hazard of readmission compared with controls. Using this threshold, we identified 28 MP cases, representing a prevalence of 0.12% of first psychiatric admissions for psychosis. Within the MP group, 13 of 28 patients were prescribed antipsychotics three months post-discharge. A non-significant trend was observed suggesting that antipsychotic use was associated with reduced readmission rates.

**Table 1.** Cox models of psychosis group with TSH and age conditions. Table 1. Long description.

Model	Variable	Coef	Exp(coef )	Se(coef )	z	p
Myxedema TSH ≥40	Psychosis group	–0.178	0.837	0.280	–0.635	0.525
Myxedema TSH ≥40, Age<60	Psychosis group	–0.513	0.599	0.365	–1.404	0.16
Myxedema TSH ≥60	Psychosis group	–0.771	0.463	0.420	–1.838	0.066
Myxedema TSH≥60, Age<60	Psychosis group	–1.270	0.281	0.587	–2.161	0.031
Myxedema TSH ≥80	Psychosis group	–1.150	0.317	0.589	–1.953	0.051
Myxedema TSH ≥80, Age<60	Psychosis group	–1.333	0.264	0.719	–1.855	0.064

**Conclusions:**

This study represents the first systematic characterization of MP within a psychiatric inpatient population. Our analysis suggested that there is a significant difference in 12-month readmission rates for cases of potential MP compared to matched controls when using criteria of TSH ≥ 60 mIU/L and age < 60. Our findings support preliminary criteria for defining MP and suggest that antipsychotics may help protect against readmission in these cases. Further research is needed to validate diagnostic thresholds and clarify optimal management strategies.

**Disclosure of Interest:**

None Declared

## Long descriptions

### Table 1. Long description

A table with seven columns: Model, Variable, Coef, Exp(coef), Se(coef), z, and p. All models analyze the Psychosis group variable.

* Myxedema T S H greater than or equal to 40: Coef -0.178, Exp(coef) 0.837, Se(coef) 0.280, z -0.635, p 0.525.

* Myxedema T S H greater than or equal to 40, Age less than 60: Coef -0.513, Exp(coef) 0.599, Se(coef) 0.365, z -1.404, p 0.16.

* Myxedema T S H greater than or equal to 60: Coef -0.771, Exp(coef) 0.463, Se(coef) 0.420, z -1.838, p 0.066.

* Myxedema T S H greater than or equal to 60, Age less than 60: Coef -1.270, Exp(coef) 0.281, Se(coef) 0.587, z -2.161, p 0.031.

* Myxedema T S H greater than or equal to 80: Coef -1.150, Exp(coef) 0.317, Se(coef) 0.589, z -1.953, p 0.051.

* Myxedema T S H greater than or equal to 80, Age less than 60: Coef -1.333, Exp(coef) 0.264, Se(coef) 0.719, z -1.855, p 0.064.

Navigate back to Table 1..

